# Association between the Polymorphisms rs2070744, 4b/a and rs1799983 of the *NOS3* Gene with Chronic Kidney Disease of Uncertain or Non-Traditional Etiology in Mexican Patients

**DOI:** 10.3390/medicina59050829

**Published:** 2023-04-24

**Authors:** Alejandro Marín-Medina, José Juan Gómez-Ramos, Norberto Mendoza-Morales, Luis Eduardo Figuera-Villanueva

**Affiliations:** 1 Departamento de Biología Molecular y Genómicas, Centro Universitario de Ciencias de la Salud (CUCS), Universidad de Guadalajara (UdeG), Guadalajara 44100, Jalisco, Mexico; 2 Departamento de Urgencias, Hospital General de Zona No. 89, Instituto Mexicano del Seguro Social, Guadalajara 44100, Jalisco, Mexico; 3 Programa de Especialización en Medicina de Urgencias, Centro Universitario de Ciencias de la Salud (CUCS), Universidad de Guadalajara (UdeG), Guadalajara 44100, Jalisco, Mexico; 4 Centro de Investigación Biomédica de Occidente (CIBO), Instituto Mexicano del Seguro Social, Guadalajara 44100, Jalisco, Mexico

**Keywords:** renal insufficiency, chronic, polymorphism, genetic, nitric oxide synthase type III

## Abstract

*Background and Objectives*: Chronic Kidney Disease of uncertain or non-traditional etiology (CKDnT) is a form of chronic kidney disease of undetermined etiology (CKDu) and is not associated with traditional risk factors. The aim of this study was to investigate the association of polymorphisms rs2070744, 4b/a and rs1799983 of the *NOS3* gene with CKDnT in Mexican patients. *Materials and Methods*: We included 105 patients with CKDnT and 90 controls. Genotyping was performed by PCR-RFLP’s, genotypic and allelic frequencies were determined and compared between the two groups using *χ*^2^ analysis, and differences were expressed as odd ratios with 95% confidence intervals (CI). Values of *p* < 0.05 were considered statistically significant. *Results:* Overall, 80% of patients were male. The rs1799983 polymorphism in NOS3 was found to be associated with CKDnT in the Mexican population (*p* = 0.006) (OR = 0.397; 95% CI, 0.192–0.817) under a dominant model. The genotype frequency was significantly different between the CKDnT and control groups (*χ*^2^ = 8.298, *p* = 0.016). *Conclusions*: The results of this study indicate that there is an association between the rs2070744 polymorphism and CKDnT in the Mexican population. This polymorphism can play an important role in the pathophysiology of CKDnT whenever there is previous endothelial dysfunction.

## 1. Introduction

Chronic kidney disease (CKD) is a multifactorial pathophysiologic irreversible process that often leads to a terminal state in which the patient requires renal replacement therapy [1].

The prevalence of the disease has been increasing worldwide. In the United States of America, the United States Renal Data System (USRDS) reported in December 2014 a prevalence of 678,383 cases, which means there were 2067 cases per million inhabitants. In Mexico, as in most of the world, there has been a marked increase in the prevalence and incidence of CKD. The USRDS reported 450 cases per million inhabitants in 2010 for the state of Jalisco [2]. “According to the latest statistics provided by the IMSS an incidence of CKD patients is estimated at 377 cases per million inhabitants, with a prevalence of 1142. Currently, there are around 52,000 patients on replacement therapy of which 80% are treated at this institution. There was an increase of 92 patients per million inhabitants from 1999 to 400 in 2008” [3].

In high-income countries, it is most associated with noncommunicable diseases, such as diabetes and hypertension. However, in low- and middle-income countries, it has several additional potential etiologies, such as infectious diseases and environmental toxins, but many remain unknown. Chronic kidney disease of uncertain or non-traditional etiology (CKDnT) is a term that has been used to describe CKD that is not attributable to any traditional risk factor and is characterized by rapid progression [4]. In some regions of Mexico and Central America there is a group of patients with CKDnT whose etiology seems to have a strong environmental component, and perhaps a genetic susceptibility of our population to the development of this disease; however, this is not entirely clear [5]. This disease has been related to the use of pesticides, recurrent episodes of dehydration and changes in the intestinal microbiota, among other environmental factors. The genetic components have been minimally studied in this disease [6,7].

This type of kidney disease is known as CKDnT, and it seems to be an emerging disease in countries such as Mexico and El Salvador, among others. In some regions of southern Mexico, a prevalence of up to 25% has been reported for this disease [8]. This disease usually occurs predominantly in young men with occupations associated with agriculture and who present CKD in advanced stages with little or no proteinuria at the time of diagnosis [9]. A similar situation has been observed in patients from other countries such as India, Brazil and Sri Lanka. However, the studies published in different regions of the world on this disease seem to have a regional scope since there are no studies that include all the factors; environmental factors have been studied separately [6].

Other studies propose the use of biomarkers in CKDnT, some with possible early utility in the development of the disease and others with predictive utility for renal recovery; however, due to the complex multifactorial etiology of the disease and the economy of the countries where this disease has a high prevalence, research and the potential clinical use of these biomarkers have been limited [10].

Very little is known about the genetic factors associated with this disease. Something that draws attention in relation to CKDnt is that it seems to occur more frequently in certain populations compared to others, so there could be some genetic susceptibility in certain populations for the development of this disease. A study in a population from India reported that some polymorphisms in the *KCNA10* (Potassium Voltage-Gated Channel Subfamily A Member 10) and *SLC13A3* (Solute Carrier Family 13 Member 3) genes are associated with a predisposition to develop CKDnT in this population [11]. We decided to study the possible role of NOS3 gene variants in the genetic predisposition of our population to develop CKDnT.

Nitric oxide (NO) is a gas with a very short half-life and is the main vasodilator at the renal level. NO has various functions related to renal and glomerular hemodynamics, but its most significant effects at this level are the promotion of diuresis and natriuresis, and to regulate the secretion of renin [12]. The enzyme nitric oxide synthase is responsible for the formation of nitric oxide. The endothelial isoform called endothelial nitric oxide synthase (eNOS) is expressed in the kidney and is encoded by the NOS3 gene located in 7q36.1. When there is kidney disease, the production of NO is lower due to a decrease in the substrate of the enzyme (L-arginine) or an increase in the bioavailability of enzyme inhibitors such as dimethylarginine (ADMA) [13].

In the *NOS3* gene, three polymorphisms including rs1799983 (also called Glu298Asp or 894G>T), VNTR (Variable Number of Tandem Repeats) in intron 4 (also called 4b/a) and rs2070744 (also called -786 T>C and located in the promoter of the gene) have been analyzed.

These polymorphisms have been associated with many diseases such as atherosclerosis, coronary spasm induced by acetylcholine, hypertension, Alzheimer’s disease, hypertensive disease of pregnancy and prostate cancer, among others [14,15]. It has been observed that the presence of these polymorphisms can decrease the biosynthesis of NO by the enzyme through different mechanisms [16,17,18,19]. It is known that endothelial dysfunction is one of the main mechanisms in the genesis and progression of diabetic nephropathy and in general of CKD. Any mechanism that alters the physiology of NO could be repeated in endothelial homeostasis and lead to a pathological state [19,20].

The objective of this study was to determine if there is an association between the polymorphisms rs1799983, VNTR in intron 4 and rs2070744 of the NOS3 gene with CKDnT in Mexican patients.

## 2. Material and Methods

### 2.1. Study Design

This was a case-controlled study designed to investigate the association between the NOS3 polymorphism with CKDnT in Mexican patients.

### 2.2. Patients and Control Samples

A total of 195 subjects, 105 with CKDnT (cases), 90 without CKDnT (controls) were included in this study.

A genomic DNA library of patients with CKDnT was used, the patients with CDKnT were selected from the Department of Nephrology of the Hospital Civil “Fray Antonio Alcalde”, a tertiary care center in Guadalajara, Mexico that receives patients from several western states of the country.

The cases were patients who had a glomerular filtration rate (GFR) of less than <15 mL/min/1.73 m^2^/SC of body surface area (BSA) (as calculated according to the CKD-EPI equation) at the time of being included in the study, of etiology not identified and not associated with other risk factors (e.g., type 2 diabetes mellitus, essential hypertension, glomerulonephritis, infection, nephrotoxic drugs, autoimmune diseases, etc.). None of the included patients had started renal replacement therapy at the time of the study. A complete medical history was obtained. All patients complied with the protocol for CKDnT required by the nephrologist.

The control group consisted of healthy individuals, without CKD (FGR > 110 (±4.7) mL/min/1.73 m^2^/SC). All the subjects included in this study were unrelated individuals, from the state of Jalisco, in western Mexico.

### 2.3. Genotyping of Polymorphisms

#### 2.3.1. rs1799983 Polymorphism

Genomic DNA was isolated from whole blood using Miller’s method [21]. The rs1799983 polymorphism was genotyped using the primers Forward 5′AAGGCAGGAGACA GTGGATGGA-3′ and Reverse 5′-CCC AGT CAA TCC CTT TGG TGCTCA -3′. The following concentrations per sample were used: buffer (10×) 1.5 µL, 1 µL primers, 1.8 µL magnesium chloride, 0.3 µL dNTP’s, 6.4 µL water, 0.05 µL Taq pol and 3 µL of genomic DNA. The amplification conditions were the following: initial denaturation 94 °C for 5 min, denaturation 94 °C for 1 minute, alignment 65 °C for 1 min, extension 72 °C for 1 min, for 35 cycles and final extension 72 °C for 7 min. A 248 base pair (bp) fragment was amplified. This fragment was subjected to digestion with the RFLP’s technique with the *Ban II* enzyme, which produces a fragment of 163 and 85 bp in the case of the G allele, and no cut occurs in the case of the T allele. The concentrations used for the digestion with the *Ban II* enzyme were as follows: buffer (10×) 1.5 μL, amplification product 3 μL, enzyme 0.1 μL, water 11.5 μL

#### 2.3.2. VNTR in Intron 4 (4b/a)

For the identification of the VNTR in intron 4, the primers Forward 5′-AGG GGG TAT GGT AGT GCC TTT-3′ and Reverse 5′-TCT CTT AGT GCT GTG GTC AC-3′ were used. The following concentrations were used: buffer (10×), 5 pmol of each primer, 200 µm of dNTPs, 1.5 mM of MgCl², 1.5 units of taq polymerase, 10% BSA (albumin acid) and 50 ng of genomic DNA. The experiment was performed under the following amplification program: denaturation for 4 min at 94 °C, 35 cycles (1 min at 94 °C, 1 min at 57 °C, and 1.30 min at 72 °C) and a final extension of 7 min at 72 °C. A fragment of 420 bp was produced in the case of allele a, and a fragment of 393 bp in case of allele b.

#### 2.3.3. rs2070744 Polymorphism

The rs2070744 polymorphism was genotyped using the primers Forward 5′-TGG AGA GTG CTG GTG TAC CCC A-3′ and Reverse 5′-GCC TCC ACC CCC ACC CTG TG-3′. The following concentrations were used: buffer (10×), 5 pmol of each primer, 200 µm of dNTPs, 1.5 mM of MgCl², 1.5 units of taq polymerase, 10% BSA (albumin acid) and 50 ng of genomic DNA. The experiment was performed under the following amplification program: denaturation for 4 min at 94 °C, 35 cycles (50 sec at 94 °C, 55 sec at 59 °C and 50 sec at 72 °C) and a final extension of 7 min at 72 °C. A 180 bp fragment was amplified, this fragment was digested with the restriction enzyme *Msp I*, cuts of 140 and 40 bp were identified in the case of the T allele and 90, 50 and 40 bp in the case of the C allele. Product identification was performed with 6–8% polyacrylamide gels. The concentrations used for the digestion with the *Msp I* enzyme were buffer (10×) 1.5 μL, amplification product 3 μL, enzyme 0.1 μL, water 11.5 μL.

## 3. Statistical Analysis

SPSS Windows, version 24 (SPSS Inc., Chicago, IL, USA) was used for the statistical analysis. Genotypes and allele frequencies were compared between groups using Pearson’s chi-square test, and odds ratios (OR) with 95% confidence intervals (CIs) were calculated. To analyze differences in genotype distributions under autosomal dominant and recessive models, Pearson’s chi-square test was used and ORs were calculated.

Genetic data were analyzed using SNPStats and Hardy–Weinberg equilibrium (HWE) was tested using Pearson’s chi-square test with a threshold of *p* < 0.05 in cases and normal controls. Quantitative data are expressed as mean ± S.D. and were compared using Pearson’s chi-square test for categorical data and Mann–Whitney U test, where applicable. Multinomial logistic regression was performed in genotype comparisons with age and gender as covariates. Values of *p* < 0.05 were considered statistically significant.

## 4. Ethical Considerations

The study was carried out in accordance with the principles of the Declaration of Helsinki, and all study participants signed an informed consent document. The project was registered with the ethics committee of the Centro de Investigacion Biomedica de Occidente (CIBO) belonging to the Instituto Mexicano del Seguro Social (IMSS) with registration number R-2015-1305-9.

## 5. Results

### 5.1. Characteristics of Patients and Controls

The study included 105 patients with CKDnT and 90 controls. The age of the subjects with CKDnT ranged from 15 to 79 years (24.06 ± 6.42), and that of controls ranged from 17 to 48 years (26.97 ± 12.36). No association was found between the distribution of polymorphisms and age in the study population (*p* = 0.812). Therefore, age does not influence the distribution of genotypes.

Around 80% of patients and 71% of controls were male. No significant association was found between the gender and the distribution of the various polymorphisms studied (*p* = 0.148) (Table 1).

### 5.2. Genotyping of NOS3 Gene Polymorphisms

The distributions of the 3 variants studied in the control group were consistent with the HWE (*χ*^2^ = 0.11, 0.70 and 1.66 for 4 b/a, rs2070744 and rs1799983, respectively); all *p* values > 0.05 (0.73, 0.40 and 0.19, respectively).

### 5.3. Association of NOS3 Gene Polymorphisms with CKDnT

NOS3 gene polymorphisms were genotyped in 105 patients and 90 controls. Table 2 shows the frequencies of genotypes and alleles of the 4 b/a genotypes in the study subjects. The “a/a” genotype was observed with very low frequency in both groups (1.1% vs. 1.9% respectively). While the “b/a” genotype was higher in the control group, the “b/b” genotype was higher in the CKDnT group. Patients and controls with the “a/a” genotype were not statistically analyzed, due to the low frequency with which it was observed. Our results indicate that the difference in the distribution of the 4b/a genotypes was not statistically significant between the two groups.

In the case of the rs2070744 polymorphism, the CC genotype was more common in patients than in controls (3.8% vs. 1.1%), and the TT genotype was more frequent in controls than in patients (86.7% vs. 78.1%) Patients and controls with the “C/C” genotype were not statistically analyzed, and the differences were not statistically significant (Table 2).

In the case of the rs1799983 polymorphism, the GG genotype was observed more frequently in the control group, and this was statistically significant (82.3% vs. 64.8%), while the TT genotype was more frequent in the CKDnT group (7.6% vs. 2.2%) and the differences were statistically significant (*p* = 0.016) (Table 2).

Multinomial logistic regressions showed that age and gender did not influence the distribution of genotypes. For rs1799983, age showed *p* = 0.824 and *p* = 0.358 for TT and GT, respectively. Gender had a *p* = 0.794 and *p* = 0.205 for TT and GT, respectively. In the case of the 4 b/a polymorphism, age showed *p* = 0.408 for a/b and *p* = 0.380 for a/a. Gender had a *p* = 0.100 for a/b, and the a/a genotype was not statistically analyzed. in the case of rs2070744, a *p* = 0.667 for CC and a *p* = 0.506 for TC were observed for age. Gender had a *p* = 0.490; CC was not statistically analyzed due to the low frequency observed.

### 5.4. Analysis of Associations of NOS3 Polymorphisms with CKDnT under Dominant and Recessive Inheritance Models

In the case of the rs1799983 polymorphism, when analyzed under a dominant model, the difference between the CKDnT and control groups was statistically significant with an *X*^2^ value of 7.465, a *p* value of 0.006 and OR (95% CI) of 0.397 (0.203–0.779). In the recessive model, no statistical significance was observed (*p* = 0.089). The frequency of the T allele was higher in CKDnT (21.42%) compared to the control group (10.55%), and the difference was statistically significant with a *X*^2^ value of 8.353, a p value of 0.0038 and OR (95% CI) of 0.433 (0.243–0.722) (Table 3). No significant differences were found in the distribution of the genotypes between the two groups for the 4 b/a polymorphism, under any model. The a allele was more frequent in patients (9.52%) compared to controls (12.22%), but no statistically significant difference was found (Table 3). In the case of the rs2070744 polymorphism, for the dominant model it was observed that both the TT genotype and the TC+CC genotype were more frequent in the CKDnT group; this difference was not statistically significant. The CC genotype was more frequent in the CKDnT group, and no significant difference was observed when the recessive model was analyzed. The minor allele (C) was more frequent in patients (12.85%) than in controls (7.78%), and no statistical significance was observed (Table 3).

## 6. Discussion

This is the first study that investigates the association between the polymorphisms rs1799983, 4b/a and rs2070744 of the NOS3 gene and CKDnT in Mexican patients.

Regarding the sex of the patients, in a study conducted in the Mexican population where patients from 21 states of the republic were included, a male/female ratio of 1:1 was reported, but in that study patients with chronic kidney disease of any etiology were included, so a report that includes only patients with CKD of unknown etiology does not exist [22]. However, several reports attribute to this disease a strong environmental component (use of agrochemicals, heavy metal contamination of water, etc.), and it could be related to the occupation of patients. This could explain why it was found that a greater number of men are affected and mostly those with occupations related to agriculture [23,24,25,26]. Therefore, the differences found in relation to gender may have a direct relationship with the social-occupational factor.

In relation to genotypic and allelic frequencies, in a study conducted in Mexican patients with diabetic nephropathy, similar genotypic and allelic frequencies were found [27]. However, these frequencies may vary depending on the population studied [28,29,30].

The role that NO may play in CKD is still not entirely clear. However, in studies carried out on different rat species, it was observed that the group of rats that showed the highest expression of the enzyme eNOS were resistant to the development of kidney disease [31]. Thus, NO seems to play an important role in renal hemodynamics.

Although the results of association studies between these variants and CKD are controversial, the evidence supports the association between these polymorphisms and the development and progression of CKD, especially with diabetic nephropathy and the variant rs1799983 [32,33,34,35]. The positive or negative association depends on the population studied.

Although other studies have already shown that there is an association between these polymorphisms and the development of kidney disease, association with parameters of renal function has also been reported in patients with CKD of a determined etiology [1,36,37,38,39]. This association is not well characterized at the molecular level and could be related to the functional effect of the polymorphisms on the enzyme. In the case of rs1799983, the presence of the Asp298 allele affects the oxidase domain of the enzyme. This domain is the binding site of the cofactor (BH4) and the substrate (L-Arginine), and this change makes the enzyme more susceptible to proteolytic degradation, thus decreasing the bioavailability of NO [40]. In a study carried out in Pakistani women who suffered from preeclampsia (a disease in which endothelial dysfunction plays a central role), it was observed that the presence of this variant modifies the flexibility of the protein. Furthermore, in this same study, variants were found in the promoter of the NOS3 gene, which modified the binding site of the transcription factors STAT 3 and 6. This seems to significantly affect the production of NO [41].

It has been demonstrated that the repeated 27 bp in the intron 4 of the NOS3 gene reduces levels of the messenger RNA of the eNOS protein, as well as the expression of the protein in transfected endothelial cells, either by alteration in the acetylation patterns of histones, or by modifying DNA methylation patterns in regions close to the repeat or close to the promoter of this gene [42].

The rs2070744 polymorphism located in the 5’ region has been associated with a decrease in the expression of the NOS3 gene [43], since it reduces the gene transcription rate by half (50%). It has been described that this variant can bind to the A1 replication protein [43,44], which is part of the RPA complex, a single-strand junction complex which is involved in DNA replication, repair and recombination [44]. In studies carried out on mice, it was observed that mice with this variant had a decrease of up to 40% in gene transcription and a significant reduction in nitrite levels [39,40,41]. This polymorphism has been associated with other diseases related to endothelial dysfunction [41], so the molecular mechanisms related to the function of NO in the vascular endothelium seem to be the same in renal endothelial dysfunction.

At the kidney level NO performs several functions related to renal and glomerular hemodynamics; its most important effects at that level are to promote diuresis and natriuresis, as well as the regulation of renin secretion [45,46].

eNOS is expressed in large amounts in the renal vascular endothelium (including afferent and efferent arterioles as well as vasa recta), in addition to being expressed in proximal ascending tubules, the thick portion of the loop of Henle and collecting ducts; however, at the level of the proximal tubules, the precise role of NO is unknown. It has been observed that in mice with little or no expression of the eNOS enzyme it increased the reabsorption of NaCl and in this way the GFR was increased, favoring the appearance of hypertension and later endothelial dysfunction [12,46,47]. Some studies conclude that these polymorphisms are only clinically important in the presence of endothelial dysfunction [13,18,19].

NO plays a very important role in cellular signaling. When it binds to the heme group, it induces a structural change in the enzyme guanylyl cyclase. This enzyme catalyzes the conversion of guanosine triphosphate (GTP) to cyclic guanosine monophosphate (cGMP). The cGMP generated is responsible for many of the physiological effects performed by NO. NO can also signal independently of cGMP, this effect is performed by nitrosilar thiol groups of cysteine residues in different intracellular proteins [48,49,50]. It has been observed that when there is renal endothelial dysfunction, NO levels fall, and this increases the expression of the arginase enzyme (possibly due to alterations in the signaling pathways) especially the isoform II, causing an increase in the production of urea [51]. Another of the metabolic pathways related to NO is the formation of creatine. It has been observed that the enzyme arginine-glycine amidino transferase can use homoarginine as a substrate. Homoarginine is a structural analogue of arginine which plays a crucial role in biochemistry of cerebrovascular accidents, and that seems to increase in situations where there is little production and/or bioavailability of nitric oxide. In this way, it increases the formation of creatine [52,53], so the presence of these polymorphisms in a renal physiology modified by the disease could modify the biochemistry at that level.

The GFR allows estimation of the renal function, it varies in each individual and is influenced by genetic and environmental factors. Although some candidate genes that seem to influence GFR have been postulated, the results are still controversial [23], and apparently variants of the NOS3 gene do not seem to influence GFR.

This study had some limitations, one of which was the small size of the sample and that we only included patients from a single hospital. However, this hospital receives patients from various states in western Mexico. The NSG (Next Generation Sequencing) method was not performed because there was no external funding, so resources were limited, in addition to the fact that this method is not routinely performed in our center. There are differences in the distribution of age and sex in the controls; however, it was observed that none of these variables affected the distribution of the genotypes. More studies are needed to analyze the influence of genetic factors on CKDnT, since these could change according to the population studied. Studies are also needed to analyze the complex interaction between genetic and environmental factors in this and other diseases, where endothelial dysfunction plays a central role.

## 7. Conclusions

We found an association between the rs2070744 polymorphism with CKDnT under a dominant model. This polymorphism can play an important role in the pathophysiology of CKDnT whenever there is previous endothelial dysfunction. Further studies with larger sample sizes, replication studies and other studies involving other genetic variants related to the biochemistry of renal function and its environmental interactions are necessary.

## Figures and Tables

**Table 1:** General characteristics of the population studied.

Variable	Values		
	Cases, Number (%)	Controls, Number (%)	
Age	26.97 (±12.36) ^a^	24.06 (±6.24) ^a^	*p* = 0.821
Sex	n	n	*X*^2^ = 2.093 *p* = 0.148
Male	84 (80)	64 (71)	
Female	21 (20)	26 (29)	
Laboratory studies			-
Urea	204.99 (± 105.52) ^b^	22.10 (± 4.5) ^b^	
Creatinine	14.73 (± 6.77) ^b^	0.5 (± 0.2) ^b^	
Parameters renal function			-
GFR	4.91 (±2.44) ^c^	110 (± 4.7) ^c^	

*p* values were derived from the *χ*^2^ test for categorical variable (gender) and Mann–Whitney U test for continuous variable (age). Abbreviations: GFR, Glomerular filtration rate. ^a^ Standard deviation; ^b^ mg/Dl; ^c^ mL/min/1.73 m^2^ /SC.

**Table 2:** Genotypic and allelic frequencies.

	Polymorphism	Cases, Number (%)	Controls, Number (%)	
**Genotypic Frequencies**	***4 b/a***			*X*^2^ = 1.712 *p* = 0.425
	a/a	2 (1.9)	1 (1.1)	
	a/b	16 (15.2)	20 (22.2)	
	b/b	87 (82.9)	69 (76.7)	
	Total	105 (100.0)	90 (100.0)	
	**rs2070744**			*X*^2^ = 3.032 *p* = 0.220
	C/C	4 (3.8)	1 (1.1)	
	T/C	19 (18.1)	11 (12.2)	
	T/T	82 (78.1)	78 (86.7)	
	Total	105 (100.0)	90 (100.0)	
	**rs1799983**			*X*^2^ = 8.298 *p* = 0.016
	T/T	8 (7.6)	2 (2.2)	
	G/T	29 (27.6)	14 (15.5)	
	G/G	68 (64.8)	74 (82.3)	
	Total	105 (100.0)	90 (100.0)	
**Allelic frequencies**	***rs1799983***			-
	G	(80)	(89)	
	T	(20)	(11)	
	***4 b/a***			-
	a	(9)	(12)	
	b	(91)	(88)	
	***rs2070744***			-
	C	(13)	(8)	
	T	(87)	(92)	

Binary Logistic Regression was tested for all three polymorphisms between groups and rs1799983 was related to CKDnT.

**Table 3:** Inheritance models for analysis of association between CKDnT and polymorphisms of the *NOS3* gene.

			Cases n(%)	Controls n(%)	
***4 b/a***	Dominant Model	bb	87 (82.85)	69 (76.67)	*X*^2^ = 1.161
		ba+bb	18 (17.50)	21 (23.33)	*p* = 0.281
					OR (95% CI) = 1.471 (0.727–2.975)
	Recessive model	ba+bb	103 (98.10)	89 (98.89)	*X*^2^ = 0.202
		aa	2 (1.90)	1 (1.11)	*p* = 0.654
					OR (95% CI) = 1.728 (0.154–19.379)
	Allele frequencies	a	20 (9.52)	22 (12.22)	*X*^2^ = 0.734
		b	190 (90.48)	158 (87.78)	*p* = 0.391
					OR (95% CI) = 1.323 (0.697–2.512)
***rs2070744***	Dominant model	TT	82 (78.10)	78 (86.67)	*X*^2^ = 2.418
		CC+TC	23 (21.90)	12 (13.33)	*p* = 0.120
					OR (95% CI) = 0.548 (0.256–1.177)
	Recessive Model	TC+TT	101 (96.20)	89 (98.88)	*X*^2^ = 1.412
		CC	4 (3.80)	1 (1.11)	*p* = 0.235
					OR (95% CI) = 3.525 (0.387–32.123)
	Allele frequencies	C	27 (12.85)	14 (7.78)	*X*^2^ = 2.658
		T	183 (87.15)	166 (92.22)	*p* = 0.103
					OR (95% CI) = 0.572 (0.290–1.127)
***rs1799983***	Dominant model	GG	68 (64.76)	74 (82.22)	*X*^2^ = 7.465
		TT+GT	37 (35.24)	16 (17.78)	*p* = 0.006
					OR (95% CI) = 0.397 (0.203–0.779)
	Recessive model	GT+GG	97 (92.38)	88 (97.77)	*X*^2^ = 2.901
		TT	8 (7.62)	2 (2.23)	*p* = 0.089
					OR (95% CI) = 3.629 (0.750–17.550)
	Allele frequencies	T	45 (21.42)	19 (10.55)	*X*^2^ = 8.353
		G	165 (78.58)	161 (89.45)	*p* = 0.0038
					OR (95% CI) = 0.433 (0.243–0.772)

Binary logistic regressions were tested for all three polymorphisms between groups. A statistically significant relationship was observed between the polymorphism rs1799983 and CKDnT under a dominant inheritance model.

## Data Availability

The data are not publicly available due to the restrictions of our institution.

## References

[1] Marín-Medina A., Brambila-Tapia A.J., Picos-Cárdenas V.J., Gallegos-Arreola M.P., Figuera L.E. (2016). eNOS gene Glu298Asp and 4b/a polymorphisms are associated with renal function parameters in Mexican patients with Fabry disease. Genet. Mol. Res..

[2] United States Renal Data System (2022). 2022 USRDS Annual Data Report: Epidemiology of Kidney Disease in the United States.

[3] Ávila-Saldívar M.N. (2013). Enfermedad renal crónica: Prevención y detección temprana en el primer nivel de atención. Med. Int. Mex..

[4] Lunyera J., Mohottige D., Von Isenburg M., Jeuland M., Patel U.D., Stanifer J.W. (2016). CKD of Uncertain Etiology: A Systematic Review. Clin. J. Am. Soc. Nephrol..

[5] Jayasumana C., Orantes C., Herrera R., Almaguer M., Lopez L., Silva L.C., Ordunez P., Siribaddana S., Gunatilake S., De Broe M.E. (2017). Chronic interstitial nephritis in agricultural communities: A worldwide epidemic with social, occupational and environmental determinants. Nephrol. Dial. Transplant..

[6] Redmon J.H., Levine K.E., Lebov J., Harrington J., Kondash A.J. (2021). A comparative review: Chronic Kidney Disease of unknown etiology (CKDu) research conducted in Latin America versus Asia. Environ. Res..

[7] Anupama P.H., Prasad N., Nzana V.B., Tiwari J.P., Mathew M., Abraham G. (2020). Dietary Management in Slowing Down the Progression of CKDu. Indian. J. Nephrol..

[8] Aguilar D.J., Madero M. (2019). Other Potential CKD Hotspots in the World: The Cases of Mexico and the United States. Semin. Nephrol..

[9] Chapman E., Haby M.M., Illanes E., Sanchez-Viamonte J., Elias V., Reveiz L. (2019). Risk factors for chronic kidney disease of non-traditional causes: A systematic review. Rev. Panam. Salud. Publica.

[10] Gunasekara T.D.K.S.C., De Silva P.M.C.S., Herath C., Siribaddana S., Siribaddana N., Jayasumana C., Jayasinghe S., Cardenas-Gonzalez M., Jayasundara N. (2020). The Utility of Novel Renal Biomarkers in Assessment of Chronic Kidney Disease of Unknown Etiology (CKDu): A Review. Int. J. Environ. Res. Public Health.

[11] Kumari R., Tiwari S., Atlani M., Anirudhan A., Goel S.K., Kumar A. (2023). Association of Single Nucleotide Polymorphisms in KCNA10 and SLC13A3 Genes with the Susceptibility to Chronic Kidney Disease of Unknown Etiology in Central Indian Patients. Biochem. Genet..

[12] Ortiz P.A., Garvin J.L. (2003). Cardiovascular and renal control in NOS-deficient mouse models. Am. J. Physiol. Regul. Integr. Comp. Physiol..

[13] Dellamea B.S., Leitão C.B., Friedman R., Canani L.H. (2014). Nitric oxide system and diabetic nephropathy. Iran. J. Otorhinolaryngol..

[14] Ryazanova M.A., Fedoseeva L.A., Ershov N.I., Efimov V.M., Markel A.L., Redina O.E. (2016). The gene-expression profile of renal medulla in ISIAH rats with inherited stress-induced arterial hypertension. BMC Genet..

[15] Szabó G.V. (2013). The role and importance of gene polymorphisms in the development of atherosclerosis. Interv. Med. Appl. Sci..

[16] Dursun H., Noyan A., Matyar S., Buyukcelik M., Soran M., Cengiz N., Seydaoglu G., Attila G., Bayazit A.K., Anarat A. (2006). Endothelial nitric oxide synthase gene intron 4 a/b VNTR polymorphism in children with APSGN. Pediatr. Nephrol..

[17] Tsukada T., Yokoyama K., Arai T., Takemoto F., Hara S., Yamada A., Kawaguchi Y., Hosoya T., Igari J. (1998). Evidence of association of the ecNOS gene polymorphism with plasma NO metabolite levels in humans. Biochem. Biophys. Res. Commun..

[18] Sofowora G., Dishy V., Xie H.G., Imamura H., Nishimi Y., Morales C.R., Morrow J.D., Kim R.B., Stein C.M., Wood A.J. (2001). In-vivo effects of Glu298Asp endothelial nitric oxide synthase polymorphism. Pharmacogenetics.

[19] El-Din Bessa S.S., Hamdy S.M. (2011). Impact of Nitric Oxide Synthase Glu298Asp Polymorphism on the Development of End-Stage Renal Disease in Type 2 Diabetic Egyptian Patients. Ren. Fail..

[20] Tiwari A.K., Prasad P.B.K.T., Kumar K.M., Ammini A.C., Gupta A., Gupta R. (2009). Oxidative stress pathway genes and chronic renal insufficiency in Asian Indians with Type 2 diabetes. J. Diabetes Complicat..

[21] Miller S.A., Dykes D.D., Polesky H.F. (1988). A simple salting out procedure for extracting DNA from human nucleated cells. Nucleic Acids Res..

[22] Méndez-Durán A., Ignorosa-Luna M.H., Pérez-Aguilar G., Rivera-Rodríguez F.J., González-Izquierdo J.J., Dávila-Torres J. (2016). Estado Actual de las Terapias Sustitutivas de la Función Renal en el Instituto Mexicano del Seguro Social. Rev. Med. Inst. Mex. Seguro. Soc..

[23] Rodríguez M.I. (2013). Sounding the alarm on chronic kidney disease in farming communities: María Isabel Rodríguez MD. Minister of health, El Salvador. Interview by Conner Gorry. MEDICC Rev..

[24] Torres C., Aragón A., González M., López I., Jakobsson K., Elinder C.G., Lundberg I., Wesseling C. (2010). Decreased kidney function of unknown cause in Nicaragua: A community-based survey. Am. J. Kidney Dis..

[25] Wesseling C., Crowe J., Hogstedt C., Jakobsson K., Lucas R., Wegman D.H. (2013). The epidemic of chronic kidney disease of unknown etiology in Mesoamerica: A call for interdisciplinary research and action. Am. J. Public Health.

[26] Jayasinghe S. (2014). La enfermedad renal crónica de etiología desconocida debe ser renombrada como nefropatía crónica por agroquímicos. MEDICC Rev..

[27] Thameem F., Puppala S., Arar N.H., Stern M.P., Blangero J., Duggirala R., Abboud H.E. (2008). Endothelial nitric oxide synthase (eNOS) gene polymorphisms and their association with type 2 diabetes-related traits in Mexican Americans. Diab. Vasc. Dis. Res..

[28] Nagase S., Suzuki H., Wang Y., Kikuchi S., Hirayama A., Ueda A., Takada K., Oteki T., Obara M., Aoyagi K. (2003). Association of eNOS gene polymorphisms with end stage renal diseases. Mol. Cell. Biochem..

[29] Ezzidi I., Mtiraoui N., Mohamed M.B., Mahjoub T., Kacem M., Almawi W.Y. (2008). Association of endothelial nitric oxide synthase Glu298Asp, 4b/a, and -786T>C gene variants with diabetic nephropathy. J. Diabetes. Complicat..

[30] Heltianu C., Costache G., Azibi K., Poenaru L., Simionescu M. (2002). Endothelial nitric oxide synthase gene polymorphisms in Fabry’s disease. Clin. Genet..

[31] Saracyn M., Czarzasta K., Brytan M., Murawski P., Lewicki S., Ząbkowski T., Zdanowski R., Cudnoch- Jędrzejewska A., Kamiński G.W., Wańkowicz Z. (2017). Role of Nitric Oxide Pathway in Development and Progression of Chronic Kidney Disease in Rats Sensitive and Resistant to its Occurrence in an Experimental Model of 5/6 Nephrectomy. Med. Sci. Monit..

[32] Kuricová K., Tanhäuserová V., Pácal L., Bartáková V., Brožová L., Jarkovský J., Kaňková K. (2013). NOS3 894G>T polymorphism is associated with progression of kidney disease and cardiovascular morbidity in type 2 diabetic patients: NOS3 as a modifier gene for diabetic nephropathy? Kidney. Blood Press. Res..

[33] Zintzaras E., Papathanasiou A.A., Stefanidis I. (2009). Endothelial nitric oxide synthase gene polymorphisms and diabetic nephropathy: A huge review and meta-analysis. Genet. Med..

[34] Zhou T.B., Xu H.L., Yin S.S. (2013). Association between endothelial nitric oxide synthase glu298asp gene polymorphism and diabetic nephropathy susceptibility. Ren. Fail..

[35] Zanchi A., Moczulski D.K., Hanna L.S., Wantman M., Warram J.H., Krolewski A.S. (2000). Risk of advanced diabetic nephropathy in type 1 diabetes is associated with endothelial nitric oxide synthase gene polymorphism. Kidney Int..

[36] Bambha K., Kim W.R., Rosen C.B., Pedersen R.A., Rys C., Kolbert C.P., Cunningham J.M., Therneau T.M. (2010). Endothelial nitric oxide synthase gene variation associated with chronic kidney disease after liver transplant. Mayo. Clin. Proc..

[37] Nath S.D., He X., Voruganti V.S., Blangero J., MacCluer J.W., Comuzzie A.G., Arar N.H., Abboud H.E., Thameem F. (2009). The 27-bp repeat polymorphism in intron 4 (27 bp-VNTR) of endothelial nitric oxide synthase (eNOS) gene is associated with albumin to creatinine ratio in Mexican Americans. Mol. Cell. Biochem..

[38] Uyar M., Sezer S., Ozdemir F.N., Kulah E., Arat Z., Atac F.B., Haberal M. (2013). Endothelial nitric oxide synthase polymorphism influences renal allograft outcome. Clin. Transplant..

[39] Casas J.P., Cavalleri G.L., Bautista L.E., Smeeth L., Humphries S.E., Hingorani A.D. (2006). Endothelial nitric oxide synthase gene polymorphisms and cardiovascular disease: A HuGE review. Am. J. Epidemiol..

[40] Zhang B., Liu Q.H., Zhou C.J., Hu M.Z., Qian H.X. (2016). Protective effect of eNOS overexpression against ischemia/reperfusion injury in small-for-size liver transplantation. Exp. Ther. Med..

[41] Shaheen G., Jahan S., Bibi N., Ullah A., Faryal R., Almajwal A., Afsar T., Al-Disi D., Abulmeaty M., Al Khuraif A.A. (2021). Association of endothelial nitric oxide synthase gene variants with preeclampsia. Reprod. Health.

[42] Zhang M.X., Zhang C., Shen Y.H., Wang J., Li X.N., Chen L., Zhang Y., Coselli J.S., Wang X.L. (2008). Effect of 27nt small RNA on endothelial nitric-oxide synthase expression. Mol. Biol. Cell..

[43] Miyamoto Y., Saito Y., Nakayama M., Shimasaki Y., Yoshimura T., Yoshimura M., Harada M., Kajiyama N., Kishimoto I., Kuwahara K. (2000). Replication protein A1 reduces transcription of the endothelial nitric oxide synthase gene containing a -786T–>C mutation associated with coronary spastic angina. Hum. Mol. Genet..

[44] Bochkarev A., Pfuetzner R.A., Edwards A.M., Frappier L. (1997). Structure of the single-stranded-DNA-binding domain of replication protein A bound to DNA. Nature.

[45] Huang P.L., Huang Z., Mashimo H., Bloch K.D., Moskowitz M.A., Bevan J.A., Fishman M.C. (1998). Hypertension in mice lacking the gene for endothelial nitric oxide synthase. Nature.

[46] Marín-Medina A., Esteban-Zubero E., Alatorre-Jiménez M.A., Alonso-Barragan S.A., López-García C.A., López-García C.A., Gómez-Ramos J.J., Santoscoy-Gutiérrez J.F., González-Castillo Z. (2018). NOS3 Polymorphisms and Chronic Kidney Disease. J. Bras. Nefrol..

[47] Shesely E.G., Maeda N., Kim H.S., Desai K.M., Krege J.H., Laubach V.E., Sherman P.A., Sessa W.C., Smithies O. (1996). Elevated blood pressures in mice lacking endothelial nitric oxide synthase. Proc. Natl. Acad. Sci. USA.

[48] Fagan K.A., Fouty B.W., Tyler R.C., Morris K.G., Hepler L.K., Sato K., LeCras T.D., Abman S.H., Weinberger H.D., Huang P.L. (1991). The pulmonary circulation of homozygous or heterozygous eNOS-null mice is hyperresponsive to mild hypoxia. J. Clin. Investig..

[49] Mujoo K., Krumenacker J.S., Murad F. (2011). Nitric oxide-cyclic GMP signaling in stem cell differentiation. Free Radic. Biol. Med..

[50] Stefano G.B., Kream R.M. (2011). Reciprocal regulation of cellular nitric oxide formation by nitric oxide synthase and nitrite reductases. Med. Sci. Monit..

[51] Zhu C., Yu Y., Montani J.P., Ming X.F., Yang Z. (2017). Arginase-I enhances vascular endothelial inflammation and senescence through eNOS-uncoupling. BMC Res. Notes.

[52] Thomas B.N., Thakur T.J., Yi L., Guindo A., Diallo D.A., Ott J. (2013). Extensive Ethnogenomic Diversity of Endothelial Nitric Oxide Synthase (eNOS) Polymorphisms. Gene Regul. Syst. Biol..

[53] Pilz S., Meinitzer A., Gaksch M., Grübler M., Verheyen N., Drechsler C., Hartaigh B.Ó., Lang F., Alesutan I., Voelkl J. (2015). Homoarginine in the renal and cardiovascular systems. Amino Acids.

